# Topological structures are consistently overestimated in functional complex networks

**DOI:** 10.1038/s41598-018-30472-z

**Published:** 2018-08-10

**Authors:** Massimiliano Zanin, Seddik Belkoura, Javier Gomez, César Alfaro, Javier Cano

**Affiliations:** 1 Centro de Tecnología Biomédica, Universidad Politéctica de Madrid, Madrid, Spain; 2 Universidade Nova de Lisboa, Lisboa, Portugal; 3 Innaxis Foundation & Research Institute, Madrid, Spain; 4 Universidad Rey Juan Carlos, Madrid, Spain; 5 University of Auckland, Auckland, New Zealand

## Abstract

Functional complex networks have meant a pivotal change in the way we understand complex systems, being the most outstanding one the human brain. These networks have classically been reconstructed using a frequentist approach that, while simple, completely disregards the uncertainty that derives from data finiteness. We provide here an alternative solution based on Bayesian inference, with link weights treated as random variables described by probability distributions, from which ensembles of networks are sampled. By using both statistical and topological considerations, we prove that the role played by links’ uncertainty is equivalent to the introduction of a random rewiring, whose omission leads to a consistent overestimation of topological structures. We further show that this bias is enhanced in short time series, suggesting the existence of a theoretical time resolution limit for obtaining reliable structures. We also propose a simple sampling process for correcting topological values obtained in frequentist networks. We finally validate these concepts through synthetic and real network examples, the latter representing the brain electrical activity of a group of people during a cognitive task.

## Introduction

Functional complex networks have brought an important advancement in the way complex systems are analysed. By shifting the focus from the underlying physical structures to the flow of information developing on top of them, functional networks yield a more detailed understanding of how, for instance, the human brain works^1,2^.

The standard way of reconstructing such representations starts with the recording of a set of time series describing the dynamics of the nodes composing the system. In neuroscience, these typically reflect the evolution of physiological observables like electric (EEG) or magnetic (MEG) fields, or the consumption of oxygen by neurons (fMRI). Afterwards, the synchronous dynamics of pairs of nodes is assessed, using various metrics spanning from linear correlations to causalities^1,3^. This approach is inherently *frequentist*, as a single value (*e.g*. the correlation coefficient) is extracted from each pair of nodes, and encoded as the weight of the corresponding link. Nevertheless, frequentist (or ‘classic’) inference is not the only alternative, as proved by the long-standing controversy with Bayesian statisticians. For decades, researchers from both fields have fiercely defended the advantages of their respective approaches, with theoretical and practical evidence supporting the superiority of the Bayesian approach and of its axiomatic and decision theoretic foundations.

The main conceptual difference between both approaches is that Bayesian inference considers data to be fixed, and the model parameters to be random, as opposed to what frequentist inference does. Furthermore, Bayesian inference—unlike frequentist—estimates a full probability model, including hypothesis testing. This entails several practical advantages, as: (*a*) incorporating prior knowledge about model parameters in a natural way; (*b*) accommodating any sample size, no matter how small; or (*c*) allowing more complex models, for which MCMC algorithms are guaranteed to converge, see^4^ for a more detailed discussion.

While Bayesian inference has previously been considered in neuroscience^5–7^, no attention has hitherto been devoted to the specific topic of functional network reconstruction. Nevertheless, in the light of the different way data and parameters are treated within both frameworks, one question arises: do observed topological metrics vary, depending on which statistical approach (frequentist *vs*. Bayesian) is applied? We demonstrate here that this is actually the case by using both statistical and topological considerations. We further show how this bias implies that topological structures are consistently overestimated in the frequentist case, since the inherent uncertainty in the observed connectivity between nodes acts as a random rewiring process. We also prove that this bias is responsible for the existence of a minimum time resolution limit, below which no network structure can reliably be estimated; and provide an efficient algorithm to reduce it.

## Frequentist vs. Bayesian Reconstruction of Functional Networks

**Figure 1:**
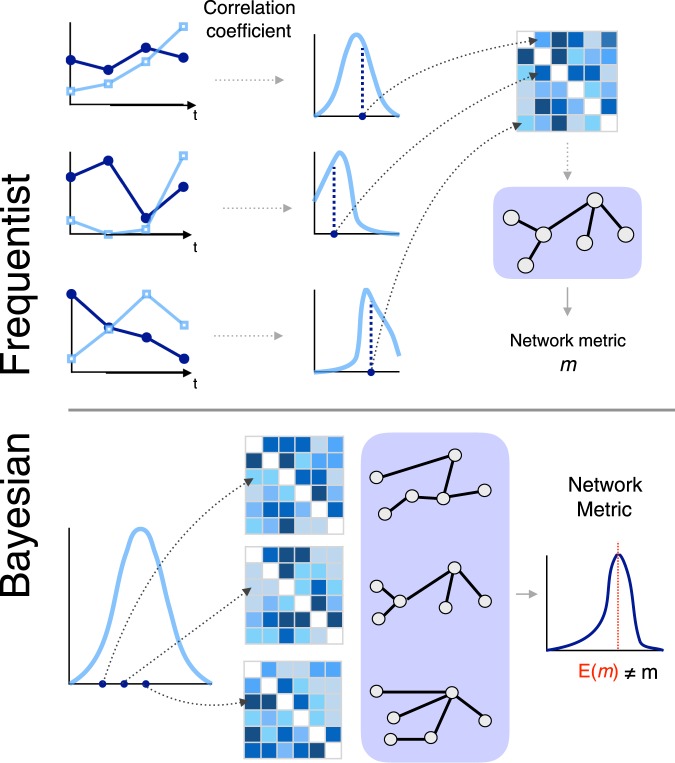
Schematic representation of the functional network reconstruction process. The top part depicts the frequentist approach, in which the classical point correlation estimate is used for each pair of time series. The bottom part represents the Bayesian counterpart, in which several weight matrices are sampled from the correlation probability distributions.

The standard procedure for network reconstruction is depicted in the upper part of Fig. 1. The starting point of the process is a set of time series, describing the dynamics of the elements composing the system under study. Denoting by *X* and *Y* any two such series—assumed, without loss of generality, to come from a bivariate normal distribution—the frequentist approach assesses their connectivity through the Pearson’s product-moment *sample* correlation coefficient *r*(*X*, *Y*) — or its absolute value |*r*(*X*, *Y*)|. Note, however, that fixing the connectivity metric does not restrict the validity of results, see Discussion. The correlation coefficients are afterwards mapped into an *adjacency matrix* ${\mathscr{A}}$ of size *N* × *N* (*N* being the number of time series, hence of nodes). Such matrix is then usually pruned, in order to delete links of low statistical significance or weight, by applying a fixed threshold or by retaining a fixed fraction of the strongest links - a step not free from problems, as shown in the literature^8,9^. Finally, a set of topological metrics is extracted from the resulting object. It is important to note that this approach implies that a point estimate *r* is used to summarise the linear dependence between *X* and *Y*. This hidden hypothesis is consistent with a frequentist approach, since it regards the correlation coefficient as a constant, estimating it through *r*(*X*, *Y*).

On the other hand, the Bayesian approach builds a full probability model for the parameters of the bivariate normal distribution the data are assumed to come from:

$$(X,Y)\sim  {\mathcal M} {\mathscr{V}}{\mathscr{N}}\,(\nu =(\lambda ,\mu ),{\rm{\Sigma }}=(\begin{array}{cc}\varphi  & \rho \\ \rho  & \psi \end{array})),$$

where *λ* = *E*[*X*], *μ* = *E*[*Y*], *ϕ* = V[*X*] and *ψ* = V[*Y*] are the means and variances of the sample data (*X*, *Y*). A prior joint distribution *p*(*λ*, *μ*, *ϕ*, *ψ*, *ρ*) can be built summarising our previous knowledge about the parameters. If we multiply it by a likelihood function based on the bivariate normal distribution, we get, *via* Bayes’ theorem, the posterior joint distribution *p*(*λ*, *μ*, *ϕ*, *ψ*, *ρ*|*X*, *Y*). After integrating it over (*λ*, *μ*, *ϕ*, *ψ*), the desired posterior marginal density *p*(*ρ*|*X*, *Y*) is obtained. A closed form expression for *p*(*ρ*|*X*, *Y*) is usually not available, except for specific choices of the prior distribution. One such case is to assume noninformative priors *p*(*λ*) ∝ 1, *p*(*μ*) ∝ 1, *p*(*ϕ*) ∝ 1/*ϕ* and *p*(*ψ*) ∝ 1/*ψ*, which imply that, for any generic prior *p*(*ρ*), the joint prior is separable, *i.e*., *p*(*λ*, *μ*, *ϕ*, *ψ*, *ρ*) ∝ *p*(*ρ*)/(*ϕψ*). Under such assumption, the posterior marginal density is then:

$$p(\rho |X,Y)\propto p(\rho )\,{(1-{\rho }^{2})}^{(n-1)/2}\,{\int }_{0}^{\infty }\,{\omega }^{-1}{(\omega +{\omega }^{-1}-2\rho r)}^{-(n-1)}\,{\rm{d}}\omega ,$$

where *r* is the (frequentist) sample correlation coefficient. Further simplifications can be made, leading to the approximate expression:

$$p(\rho |X,Y)\propto p(\rho )\frac{{(1-{\rho }^{2})}^{(n-1)/2}}{{(1-\rho r)}^{n-3/2}},$$

see^10^ for details.

Once the posterior distribution has been obtained, either in explicit form or approximated through *e.g*. a Monte Carlo estimation^11^, we can compute Bayesian credible intervals (*ρ*_*l*_,*ρ*_*u*_) for *p*(*ρ*|*X*, *Y*) to summarise our uncertainty about the correlation coefficient. Credible intervals are preferred over classical confidence intervals, since they make use of all available information (data and prior beliefs) to provide a range of possible parameter values covering some specified probability. However, under certain simplifying assumptions, it can be shown that both approaches provide similar results. Therefore, should the computational burden becomes excessive, we could rely on the usually cheaper classical solution, see Section *Correcting frequentist networks through rewiring*.

Disregarding such probability distribution, which is a measure of the uncertainty in the connectivity, is readily expected to introduce biases in the obtained results. In general terms, the extraction of a metric *m* can be seen as the application of a highly complex and non-linear function of the adjacency matrix, $m=f({\mathscr{A}})$. When it comes to compute the expected value of the function of a given random variable *X*, it is well-known that, in general, *E*[*f*(*X*)] ≠ *f*(*E*[*X*]), being the latter the wrong way to do it. Specifically, if one has a sample *x* = (*x*_1_, *x*_2_, …, *x*_*n*_), the right method to compute *E*[*f*(*x*)] implies evaluating *f* for all the elements in the sample, *i.e*. (*f*(*x*_1_), *f*(*x*_2_), …, *f*(*x*_*n*_)), and finally averaging such values. The incorrect way would calculate the sample mean $\bar{x}=E[x]$ first, and evaluate $f(\bar{x})$ afterwards. As depicted in the bottom part of Fig. 1, the correct procedure for reconstructing functional networks thus entails: (1) sampling different values of the link weights, according to the posterior distribution of *ρ*|*data*; (2) creating multiple networks, one for each sampled weight set; (3) computing the corresponding target metric *m* for each network; (4) obtaining the empirical probability distribution of the target metric *m*; and (5) calculating its expected value *E*[*m*].

**Figure 2:**
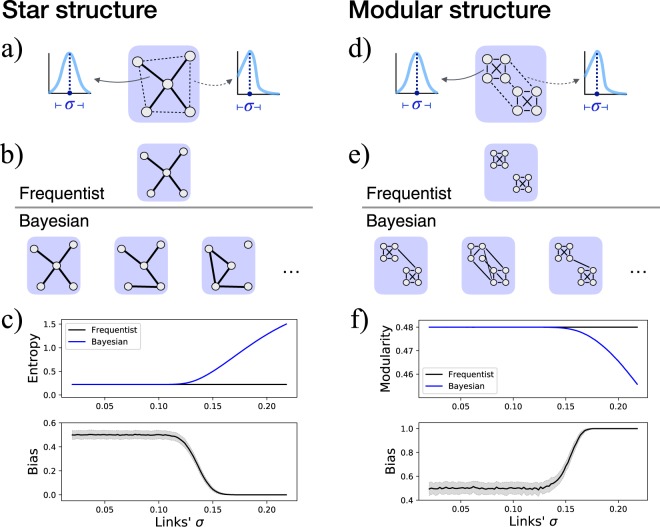
Example of the analysis of two structures, a star-like (left part) and a modular one (right part). (**a**) Initial star structure, with four nodes strongly connected with a central one, and loosely connected between them. Note that the correlation between the time series of each pair of nodes is defined with an uncertainty *σ*. (**b**) Frequentist and Bayesian results. In the former case the output is a constant network, as uncertainty is disregarded; in the latter, and due to the inherent uncertainty, different structures are generated, some of them different from the original one. (**c**) Evolution of the entropy and of the bias (see Methods for definitions) as a function of the links’ uncertainty *σ*. (**d**–**f**) Results of equivalent analyses for the modular structure.

Beyond this statistical consideration, the error made by the frequentist approach can also be understood from a topological point of view. Let us suppose one is analysing a star-like network, as depicted in Fig. 2(a), or a modular graph, as in Fig. 2(d). Additionally, let us suppose that the correlation between the time series of pairs of sensors is characterised by an uncertainty, here denoted by *σ*, which is easily depicted as the spread of the probability distribution of the correlation. As in the first case the strongest links are those connected with the central hub, and since the frequentist approach disregards their associated uncertainty, the result of the reconstruction process would always be constant: a well-defined star-like structure —see Fig. 2(b) Top. Similarly, in the second example the frequentist approach would always yield a well-defined modular structure, as depicted in Fig. 2(e) Top. On the other hand, the Bayesian approach recognises that silent links actually have non-null weights—as described by their posterior probability distribution *ρ*|*data*—albeit with lower expected values than active ones. When link weights are eventually sampled from the corresponding distribution, links between peripheral nodes may actually have greater weights than the central ones. The result is a set of networks in which the star-like and the modular structures are sometimes lost—Fig. 2(b,e) Bottom. If one then analyses the resulting structure—through *e.g*. the entropy of the degree distribution—two completely different results are found: a low constant entropy in the frequentist case, and a variable and higher value in the Bayesian case, see Fig. 2(c). One can additionally calculate a *bias*, *i.e*. a metric defined as the fraction of times the topological metric observed in the Bayesian network is smaller than what is observed in the frequentist one. Small bias’ values thus indicate that the frequentist approach is underestimating the metric (as is the case in Fig. 2(c)), large values that it is overestimating it (Fig. 2(f)), while values close to 0.5 would indicate that both approaches yield equivalent results (see Methods for further details).

Both Figs 1 and 2 suggest two important conclusions. First, that disregarding the inherent uncertainty associated with functional links introduces a bias in the obtained topological metrics. Secondly, that the link uncertainty acts like a random *rewiring process*, such that dismissing it overestimates the regularities observed in the network.

## Application to Brain Physiological Data

In order to illustrate how the previously defined bias may affect the analysis of real-world networks, we consider here a large set of functional networks representing brain dynamics in control and alcoholic subjects—see Methods for details on the data set.

**Figure 3:**
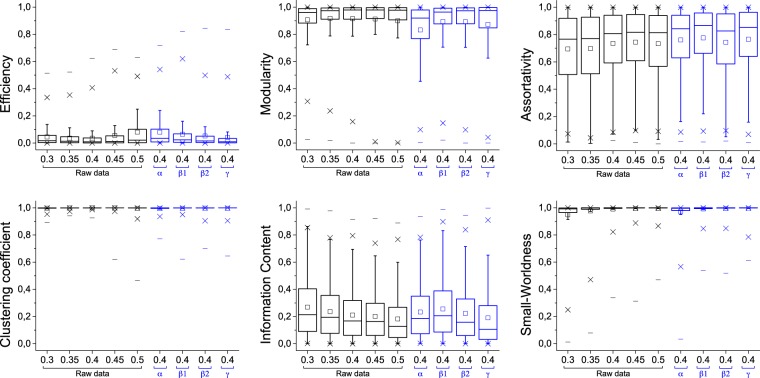
Distribution of the bias between frequentist and Bayesian values in real EEG networks, for the six considered topological metrics—see Methods for definitions and experimental data description. Each box plot corresponds to networks obtained with a fixed link density (from 0.3 to 0.5) and filtered by four frequency bands. Central horizontal bars and squares represent the median and the mean of the distribution, respectively; boxes and crosses the 25th–75th and 1th–99th percentiles; and the external horizontal lines the minimum and maximum.

Figure 3 depicts the distribution of the bias observed in six metrics commonly used in complex network, see Methods. Networks have been reconstructed using different criteria, including five different link densities in the binarisation process, and five band filtering (*i.e*. the raw time series, and bands *α*, *β*1, *β*2, and *γ*). It can be appreciated that the frequentist value overestimate the metrics in all cases, except for the Efficiency^12^ and the Information Content^13^. However, this is consistent with the insights of Fig. 2, which indicates that the rewiring introduced by the Bayesian method implies that all topological metrics are overestimated by the frequentist approach. The two exceptions—Efficiency and Information Content—are explained by the fact that such metrics are actually maximal for random networks.

Of special relevance is the analysis of the behaviour of the Small-Worldness, a metric assessing the coexistence of a high number of triangles with short geodesic distances^14^. In spite of some critical voices^15,16^, Small-Worldness has been considered as one of the landmarks of brain functional networks, describing its capacity for the simultaneous local integration and long-range transmission of information^17,18^. Beyond physiological^16^ or methodological^19^ reasons that may bias the observed Small-Worldness, we have shown that this property is also affected by the use of a frequentist approach. Figure 3 suggests that the small-world nature of the human brain should be taken with caution: even if the brain seems to possess such feature, the actual value may have been substantially overestimated.

## Time Series Length

The selection of the optimal time series length for reconstructing functional networks is, in general, a non-trivial problem, particularly complex in neuroscience. If, on one hand, long time series may seem desirable for a better estimation of functional connectivity, this should be balanced, on the other hand, by the need of a stationary dynamics. In other words, if a given cognitive task is executed in around one second, this is the maximum length that can be considered without introducing spurious information. This issue has recently been studied in, for instance^20^, finding that the time series length has profound effects in the observed topological metrics.

On top of any physiological consideration, we note here that shorter time series imply higher uncertainty in the estimation of the connectivity metric—the correlation coefficient in our case. This can be better understood by taking the star-like structure of Fig. 2 as an example. Suppose that the real structure driving the system’s dynamics has a star-like topology; and that, when calculated using the frequentist approach, the resulting functional network matches exactly the real one. By acknowledging the inherent randomness of the links’ weights, the Bayesian approach would suggest that the star-like structure is not the only possible one, but (eventually, given small enough uncertainties) just the most probable one. In other words, if the length of the used time series is not enough to ensure a small uncertainty in the links’ *σ*, results yielded by the frequentist approach cannot be trusted, even if actually correct.

**Figure 4:**
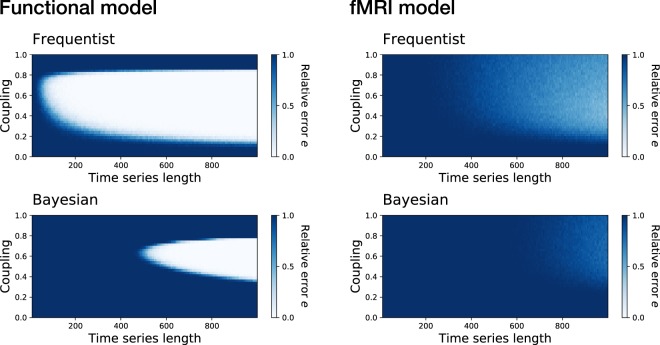
Impact of the time series length in the topological uncertainty. Evolution of the relative error *e* in the estimation of the topology, as a function of the coupling constant *γ* and the time series length, for the frequentist (Top) and Bayesian (Bottom) approaches, using a synthetic functional model (Left) and a fMRI model (Right) —see Material and Methods for definitions.

This issue is studied in Fig. 4, which reports the results obtained with two synthetic models—see Methods for a description. The four panels depict the evolution of the fraction of functional links wrongly observed in the reconstructed networks, as compared to the true connectivity, as a function of the number of data points and the coupling strength. In the left case, corresponding to a simple correlation between time series, the top blue region of both panels indicates that high couplings lead to an over-synchronisation of the system, and thus to the observation of a spurious all-to-all connectivity. A more interesting behaviour emerges for intermediate couplings (*γ* ≈ 0.5): while both methods converge towards the correct topology, the Bayesian approach requires substantially longer time series to reach the same precision. This result tells us that it is possible for the frequentist approach to detect the real functional structure, provided the information encoded in the data is explicit enough—as it has been shown in this tailored example. On the other hand, the Bayesian approach requires longer time series to resolve the topology, *i.e*. to reduce the uncertainty associated with the correlation between pairs of nodes, until a stable structure is reached. Similar conclusions can be drawn from the right panels, depicting a more complex coupling between the time series, designed to emulate the typical result of a functional Magnetic Resonance Imaging (fMRI) analysis^21^.

Summarising, the Bayesian approach always reminds that different (real) alternative connectivity patterns could yield time series that are compatible with the observed frequentist functional connectivity. In order to resolve this multiplicity in solutions, longer time series—implying a reduction in the link uncertainty—should be considered. Therefore, even if metrics estimated through the frequentist approach may *prima facie* seem more accurate, there is no guarantee that they really reflect the actual underlying topology. Frequentist results should therefore not be taken at face value, especially in real-world analyses, as there are uncountable, *a priori* unknown, situations where they may just be stemming from biases.

## Correcting Frequentist Networks Through Rewiring

The Bayesian approach yields a more complete and theoretically correct view of the system under study; this, however, comes at some costs. First, a Bayesian version may not be available for many connectivity metrics. But even if so, the associated computational burden may be prohibitive in large-scale studies. We provide here an affordable alternative, based on the creation of a set of rewired networks simulating the Bayesian output.

Let us start with the case when a complex network has been obtained using the frequentist methodology, *i.e*. when an adjacency matrix as the one in Fig. 1 Top has been calculated. Instead of using the fully Bayesian approach to obtain the probability distribution associated with each link, we propose here an alternative low-cost and efficient procedure, based on the Fisher’s transformation of the correlation coefficient, *Z*(*ρ*) = arctanh(*ρ*), see^22,23^. It is well-known that *Z*(*ρ*) follows approximately a normal distribution with standard deviation ${\sigma }_{Z}=\mathrm{1/}\sqrt{df-3}$, where *df* is the effective number of degrees of freedom, which coincides with the series length *n* if data are independent. In case of autocorrelated time series, as the ones considered here, an effective number of degrees of freedom has to be defined as:

$$\frac{1}{df}\approx \frac{1}{n}+\frac{2}{n}\,\sum _{\tau }\,{\varrho }_{ii}(\tau )\,{\varrho }_{jj}(\tau ),$$

where ${\varrho }_{xx}(\tau )$ is the autocorrelation of signal *x* at lag *τ*, see^24^ for details. We can then invert the transformation, and assume that *ρ* can be reasonably described through a normal distribution ${\mathscr{N}}\,[r,\,\tanh ({\sigma }_{Z})]$. Afterwards, an ensemble of synthetic networks is created, sampling the weight of each link from the corresponding distribution, and applying a threshold to recover a network with the same link density as the original frequentist one. Provided this approximation is good enough, calculating topological metrics on this ensemble is equivalent to computing them on an set of networks created using the Bayesian approach. Additionally, this approach can be applied with any connectivity metric, provided it can be described by a known probability function.

**Figure 5:**
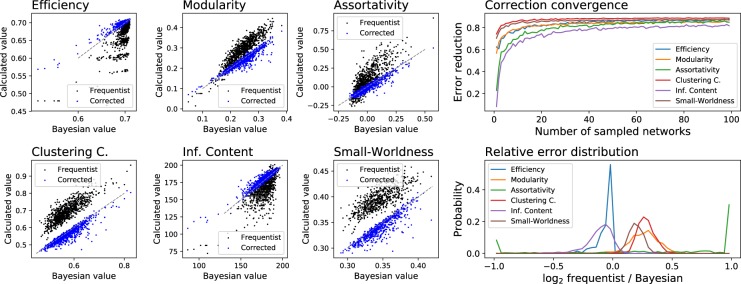
(Left panels) Comparison of Bayesian, frequentist and corrected frequentist topological values (see main text for a definition of the latter), for the EEG data set described in Methods. (Right top panel) Evolution of the reduction in the error, defined as the fraction of the error incurred by the frequentist approach disappearing after the synthetic correction, as a function of the number of sampled synthetic networks. (Right bottom panel) Probability distribution of the relative error associated with the frequentist approach, defined as the log_2_ of the ratio between the values of the frequentist and Bayesian topological metrics.

To demonstrate the effectiveness of this correction method, Fig. 5 reports six scatter plots, one for each considered topological metric, comparing the Bayesian, frequentist and corrected frequentist values for the same networks analysed in Fig. 3. It can be appreciated that the latter is an excellent approximation of the Bayesian process, while still saving orders of magnitude of computational cost. Additionally, Fig. 5 Top Right shows the evolution of the fraction of the recovered error as a function of the number of drawn synthetic networks. As we can observe, the frequentist bias can be reduced by around 80% with as low as 100 realisations.

## Discussion

The fact that the existence and strength of links in functional networks cannot assuredly be defined has profound implications in the topological analysis. As opposed to the classical frequentist point of view, we presented here a Bayesian approach, and demonstrated its theoretical advantages and capacity to account for the links’ inherent uncertainty. We have shown that, in general, reconstructing functional networks using the frequentist methodology overestimates the presence of regularities and non-trivial (*i.e*. non-random) structures. As shown in Fig. 5 Bottom Right, the Clustering Coefficient and the Modularity were overestimated, on average, by 19%, and the Small-Worldness by 13%. We further proved that such drawback is aggravated by the use of short time series, although it is possible to (partially) correct it by sampling synthetic networks. In synthesis, the scientific community, and especially the neuroscience one, has already been trying to define what a “correct” connectivity estimation really is, by proposing rules about the choice of connectivity metrics, binarization thresholds^8,9^, and time series length^20^. We nevertheless here show that an additional ingredient is essential for obtaining meaningful structures: the uncertainty in the estimation of connectivity. When such uncertainty is disregarded, for instance by resorting to a frequentist approach, potentially wrong topological structures may be detected. Note that topological metrics are not more accurate when calculated using the Bayesian approach, but they better reflect the reality of the data.

The topological bias here described is a general phenomenon, independent on the actual synchronisation or connectivity metric used, and on the data set considered. Data cannot represent the whole universe, and when coming from real observations they are usually polluted by noise. Therefore, any metric based on them is inherently uncertain and fuzzy, and topological biases, as the one we have shown here, will always appear to a greater or lesser degree. This effect is to be expected in the analysis of any real-world system in which functional representations are relevant, as *e.g*. financial markets^25^, medicine^26^, or companies’^27^ and social networks^28^.

If results presented in all research works analysing functional networks are potentially biased, this does not mean their conclusions are *de facto* wrong. For instance, while the Small-Worldness of brain functional networks may have been overestimated, to such a point that their actual value cannot be trusted, the existence of a positive value still suggests that the brain has a small-world structure—even if less marked. Additionally, functional networks corresponding, for instance, to different diseases, can still be compared, provided the respective uncertainties (and hence, the time series length) are similar.

## Methods

### Topological metrics

For the sake of completeness, we briefly describe here the topological metrics considered in this work. For more thorough definitions, the reader can consult the corresponding references, or the many reviews available in the literature^29,30^.

#### Efficiency

Measure of how efficiently information can be transmitted in a network, and defined as the inverse of the harmonic mean of the geodesic distance between nodes^12^:

1 $$E=\frac{1}{n(n-1)}\,\sum _{i,j\ne i}\,\frac{1}{{d}_{i,j}}.$$

#### Modularity

Presence of communities, *i.e*. groups of nodes more connected between them than with the remainder of the network^31^. The modularity has here been estimated through the Louvain algorithm, as proposed in^32^.

#### Assortativity

Conditional probability *P*(*k*′|*k*) that a link from a node of degree *k* points to a node of degree *k*′. It is calculated as the Pearson correlation coefficient of the degrees at either ends of a link.

#### Clustering coefficient

Measure of the presence of triangles in the network, calculated as the relationship between the number of triangles in the network and the number of connected triples.

#### Information Content

Measure assessing the presence of meso-scale structures in complex networks, based on the identification of regular patterns in the adjacency matrix of the network, and on the calculation of the quantity of information lost when pairs of nodes are iteratively merged^13^.

#### Small-Worldness

Metric capturing the degree of *Small-Worldness* of a network, defined as the coexistence of a high Clustering Coefficient and a low mean geodesic distance^14^.

#### Entropy of the degree distribution

Metric measuring the heterogeneity, in terms of Shannon’s entropy, of the distribution created by nodes’ degree^33^:

2 $$H=-\,\sum _{k}\,p(k)\,{\mathrm{log}}_{2}\,p(k).$$

The minimum *H* = 0 indicates a constant degree across all nodes, while higher values a more uniform distribution of degrees.

### EEG brain recordings

As an example of application of the proposed methodology, we considered a data set of EEG recordings from a group of alcoholic subjects and matched controls^34,35^, freely available at https://archive.ics.uci.edu/ml/datasets/EEG+Database. Each trial corresponds to an object recognition task, as described in^36^; and its corresponding EEG activity has been recorded during one second, with a 256 Hz (3.9-ms/epoch) sampling rate from 64 electrodes located at standard scalp sites. A total of 900 trials were analysed, half of them from control subjects, the remainder from alcoholic. Along with the raw time series, different filtering were considered, corresponding to the bands *α* (8.0–13.0 Hz), *β*1 (13.0–20.0 Hz), *β*2 (20.0–30.0 Hz) and *γ* (30.0–50.0 Hz).

### Bias calculation

For each set of time series, the frequentist method entails calculating one single functional network, and, from it, a (single valued) topological metric. On the other hand, the Bayesian approach entails sampling a large number of networks, each one with a potentially different topology, from which we extract the probability distribution associated with the topological metric. Specifically, we have used 10^5^ Bayesian networks in our experiments, thus yielding 10^5^ metric values.

The bias introduced by the frequentist approach is then evaluated by calculating the proportion of sampled (Bayesian) values that are smaller than the frequentist one. Note that a bias of 0.5 indicates that the frequentist value coincide with the median of the Bayesian distribution, and thus that both approaches are equivalent; while values close to 0.0 or 1.0 indicate a frequentist under- and overestimation of the metric, respectively.

### Synthetic functional network model

We simulate a system composed of *n* = 10 elements, connected according to a star-like structure, whose adjacency matrix is:

3 $${\mathscr{A}}=[\begin{array}{cccc}0 & 1 & \ldots  & 1\\ 1 & \ddots  &  & \\ \vdots  &  & 0 & \\ 1 &  &  & \ddots \end{array}]$$

The output of each element is initially a random number sampled from a normal distribution ${\mathscr{N}}(0,1)$. Afterwards, such numbers are coupled according to:

4 $${x}_{i}(t+1)=(1-\gamma ){x}_{i}(t)+\frac{\gamma }{{\sum }_{j\ne i}{a}_{j,i}}\,\sum _{j\ne i}\,{a}_{j,i}{x}_{j}(t),$$

where 0 ≤ *γ* ≤ 1 is the coupling constant. The resulting time series are then used to reconstruct the observed functional network ${\mathcal F}$; and the relative error between ${\mathcal F}$ and the original ${\mathscr{A}}$ is defined as:

5 $$e=\frac{1}{n(n-1)}\,\sum _{i,j}\,|{a}_{i,j}-{f}_{i,j}|.$$

### fMRI network model

Similarly to what was described in the previous synthetic model, we here consider a system composed of *n* = 10 elements, connected according to the star-like structure defined in Eq. 3. In order to analyse a dynamics more similar to what is observed in neuroscience studies, each element emulates the typical signal recorded by function Magnetic Resonance Imaging (fMRI) in the human brain.

The output of each element is created by firstly generating coupled time series through a VAR model and additive Gaussian noise, according to the coupling rule of Eq. 3. Subsequently, each time series is convoluted through the canonical hemodynamic response function (HRF), describing the blood and nutrient requirements of neurons as a function of their activity, and defined as a mixture of two gamma functions. As a final step, time series are down-sampled, to better reflect the technical limitations of fMRI machines. For a full definition of this approach, please refer to^37,38^.

As in the previous model, the resulting time series are used to reconstruct the observed functional network and the associated topological error *e*.
